# Risk factors for postoperative bleeding following endoscopic submucosal dissection in early gastric cancer: A systematic review and meta-analysis

**DOI:** 10.1097/MD.0000000000037762

**Published:** 2024-04-12

**Authors:** Yuanbo Gu, Shuchang Zhao

**Affiliations:** a Department of Gastroenterology, Jilin Chemical Hospital, Jilin, China.

**Keywords:** early gastric cancer, endoscopic submucosal dissection, meta-analysis, postoperative bleeding, risk factors

## Abstract

**Background:**

Early gastric cancer (EGC) presents a significant challenge in surgical management, particularly concerning postoperative bleeding following endoscopic submucosal dissection. Understanding the risk factors associated with postoperative bleeding is crucial for improving patient outcomes.

**Methods:**

Adhering to the Preferred Reporting Items for Systematic Reviews and Meta-Analyses guidelines, a systematic review and meta-analysis were conducted across PubMed, Embase, Web of Science, and the Cochrane Library without publication date restrictions. The inclusion criteria encompassed observational studies and randomized controlled trials focusing on EGC patients undergoing endoscopic submucosal dissection and their risk factors for postoperative bleeding. The Newcastle-Ottawa Scale was utilized for quality assessment. The effect size was calculated using random or fixed-effects models based on the observed heterogeneity. We assessed the heterogeneity between studies and conducted a sensitivity analysis.

**Results:**

In our meta-analysis, 6 studies involving 4868 EGC cases were analyzed. The risk of postoperative bleeding was notably increased with intraoperative ulcer detection (odds ratio: 1.97, 95% confidence interval [CI]: 1.03–3.76, *I*^2^ = 61.0%, *P* = .025) and antithrombotic medication use (odds ratio: 2.02, 95% CI: 1.16–3.51, *I*^2^ = 57.2%, *P* = .039). Lesion resection size showed a significant mean difference (5.16, 95% CI: 2.97–7.98, *P* < .01), and longer intraoperative procedure time was associated with increased bleeding risk (mean difference: 11.69 minutes, 95% CI: 1.82–26.20, *P* < .05). Sensitivity analysis affirmed the robustness of these findings, and publication bias assessment indicated no significant bias.

**Conclusions:**

In EGC treatment, the risk of post-endoscopic submucosal dissection bleeding is intricately linked to factors like intraoperative ulcer detection, antithrombotic medication use, the extent of lesion resection, and the length of the surgical procedure. These interwoven risk factors necessitate careful consideration and integrated management strategies to enhance patient outcomes and safety in EGC surgeries.

## 1. Introduction

Gastric cancer remains one of the most challenging malignancies in the medical field, with its prevalence and mortality rates making it a critical global health issue. Despite advances in diagnosis and treatment, gastric cancer often remains undetected until advanced stages, underscoring the imperative for early detection and intervention.^[1,2]^ The early detection of gastric cancer is crucial, as it significantly improves prognosis and survival rates. Recent advances in diagnostic techniques, particularly endoscopy, have enhanced the ability to detect gastric neoplasms at an earlier stage. This early detection is pivotal in improving patient outcomes and reducing the burden of this disease.

One of the landmark advancements in the treatment of early gastric cancer (EGC) is the development of endoscopic submucosal dissection (ESD). ESD is a minimally invasive technique that allows for the precise and complete removal of early-stage tumors.^[3,4]^ This technique has revolutionized the approach to EGC, offering a less invasive alternative to traditional surgical methods, with comparable efficacy and fewer complications. However, ESD is not without its challenges, with postoperative bleeding (post-endoscopic submucosal dissection bleeding, PEB) being one of the most significant concerns.^[5,6]^ PEB can lead to serious complications, including prolonged hospitalization, increased need for medical interventions, and in some cases, significant morbidity. Understanding the risk factors associated with PEB is therefore of paramount importance for improving patient care and outcomes. The causative factors for PEB are diverse and multifaceted, encompassing patient demographics, tumor characteristics, and technical aspects of the ESD procedure.^[7]^ These factors include, but are not limited to, the patient’s age, comorbid conditions, the location and size of the tumor, and the experience and technique of the endoscopist. A thorough understanding of these risk factors is essential for the development of strategies to mitigate the risk of PEB.

In light of the significant impact of postoperative bleeding (POB) on patient outcomes following ESD for EGC, the existing literature has indeed reported on various risk factors associated with POB. However, despite the wealth of individual studies and reports, there remains a conspicuous gap in the form of a comprehensive meta-analysis or systematic review that synthesizes these findings. This oversight highlights a critical need for a consolidated analysis that not only brings together these disparate pieces of evidence but also quantitatively evaluates the significance of different risk factors. Our study addresses this gap by employing rigorous systematic review and meta-analysis methods to identify, appraise, and synthesize the available evidence. By doing so, we aim to provide a robust and comprehensive assessment of the risk factors for POB in EGC patients undergoing ESD, thereby offering valuable insights for clinical practice and guiding future research endeavors in this domain.

## 2. Materials and methods

### 2.1. Search strategy

In conducting the meta-analysis, we strictly adhered to the Preferred Reporting Items for Systematic Reviews and Meta-Analyses guidelines.^[8]^ The search was executed on September 19, 2023, across 4 major electronic databases: PubMed, Embase, Web of Science, and the Cochrane Library. We set no restrictions on the publication date of the studies. The search strategy incorporated a comprehensive set of key terms, including “early gastric cancer,” “endoscopic submucosal dissection,” “postoperative bleeding,” “risk factors,” “esd complications,” “gastric neoplasms,” “post-esd bleeding,” and “hemorrhage.” Language barriers were not imposed, allowing for the inclusion of studies published in any language. Additionally, to ensure thoroughness and to capture any potentially relevant studies missed in the electronic search, we manually scrutinized the reference lists of all included articles.

### 2.2. Inclusion criteria and exclusion criteria

#### 2.2.1. Inclusion criteria.

1) *Study design*: We included observational studies (cohort, case-control, and cross-sectional studies) and randomized controlled trials that investigated the risk factors for postoperative bleeding following endoscopic submucosal dissection in patients with early gastric cancer.
2) *Population*: Studies involving patients diagnosed with early gastric cancer who underwent ESD were considered.
3) *Outcome measures*: The primary outcome of interest was postoperative bleeding following ESD. Studies needed to provide clear definitions and methods for assessing postoperative bleeding.
4) *Data reporting*: Studies had to provide sufficient data to calculate the odds ratio, relative risk, or other relevant statistics for at least 1 risk factor associated with postoperative bleeding.

#### 2.2.2. Exclusion criteria.

1) *Non-relevant populations*: Studies focusing on patients with gastric conditions other than early gastric cancer, or those not undergoing ESD, were excluded.
2) *Insufficient data*: Studies that did not report specific outcomes related to postoperative bleeding or lacked sufficient data for extraction were excluded.
3) *Review articles and editorials*: Narrative reviews, systematic reviews, meta-analyses, editorials, and opinion pieces were excluded from the primary analysis but were used for identifying additional references.
4) *Duplicate publications*: Studies reporting duplicated data or overlapping patient cohorts were excluded to avoid duplication of information.

### 2.3. Data extraction

In our systematic review and meta-analysis focused on identifying risk factors for postoperative bleeding following endoscopic submucosal dissection in early gastric cancer, we implemented a meticulous data extraction process. This process was conducted independently by 2 reviewers to ensure accuracy and objectivity. They meticulously screened the literature and extracted relevant data, with any discrepancies encountered during this phase being resolved through discussion. If a consensus could not be reached, a third-party reviewer was consulted to adjudicate the disagreement. The extracted data encompassed various essential details, including the author(s) of each study, the publication year, and the number of cases analyzed. Additionally, specific attention was given to extracting data on sample size and risk factors for postoperative bleeding. These risk factors comprised gender, intraoperative discovery of ulcers, use of antithrombotic drugs, tumor location, presence of hypertension, duration of the procedure, size of the lesion resected, and depth of infiltration. In instances where the published reports lacked certain pertinent data, we took the initiative to directly contact the original investigators via email. This proactive approach was aimed at acquiring any unpublished data that could contribute significantly to our comprehensive analysis of the risk factors associated with postoperative bleeding in early gastric cancer patients undergoing ESD.

### 2.4. Quality assessment

To ensure the reliability and validity of our meta-analysis focusing on risk factors for postoperative bleeding following endoscopic submucosal dissection in early gastric cancer, we conducted a thorough quality assessment of all included studies. This assessment was performed independently by 2 reviewers to minimize bias and ensure consistency. For the quality evaluation of the studies, we utilized the Newcastle-Ottawa Scale (NOS).^[9]^ This scale is recognized for its robustness in assessing the quality of non-randomized studies in meta-analyses. The NOS framework evaluates studies across 3 pivotal domains: selection of study groups, comparability of groups, and the ascertainment of either the exposure or outcome of interest for case-control or cohort studies, respectively. Each study was scrutinized based on these criteria and subsequently assigned a score ranging between 0 and 9, reflecting its overall quality. The scoring interpretation was as follows: studies scoring from 0 to 3 were categorized as low quality, indicating potential for significant bias; those with scores between 4 and 6 were classified as moderate quality, suggesting some bias but generally reliable findings; and studies achieving scores between 7 and 9 were considered high quality, indicating robust methodology with minimal bias.

### 2.5. Statistical analyses

We assessed the heterogeneity among the included studies using chi-square statistics, with the degree of heterogeneity quantified by the *I*^2^ value. When the *I*^2^ value was found to be less than 50% and the corresponding *P* value was .10 or higher, it was inferred that there was no significant heterogeneity among the studies. Under these circumstances, we utilized a fixed-effect model to calculate the combined effect size. Conversely, when the *I*^2^ value reached 50% or more, or the corresponding *P* value fell below .10, this indicated significant heterogeneity. In cases where only statistical heterogeneity was present, a random-effects model was applied to determine the combined effect size. To ensure the reliability of our findings, we conducted sensitivity analyses. This involved sequentially omitting each study from the meta-analysis and recalculating the overall effect size, allowing us to identify any potential sources of heterogeneity and to assess the robustness of our results. To evaluate the presence of potential publication bias, we analyzed the symmetry of the funnel plot. An even distribution of data points around the apex of the funnel plot would suggest a lower likelihood of the results being influenced by publication bias. Additionally, we utilized Egger’s linear regression test as a quantitative tool to further investigate the presence of publication bias. All statistical tests were 2-sided, and a *P* value of less than .05 was considered to indicate statistical significance. The data analysis was carried out using Stata version 17 (StataCorp, College Station, TX), ensuring a rigorous and comprehensive statistical evaluation of the data.

## 3. Results

### 3.1. Search results and study selection for meta-analysis

**Figure 1.**
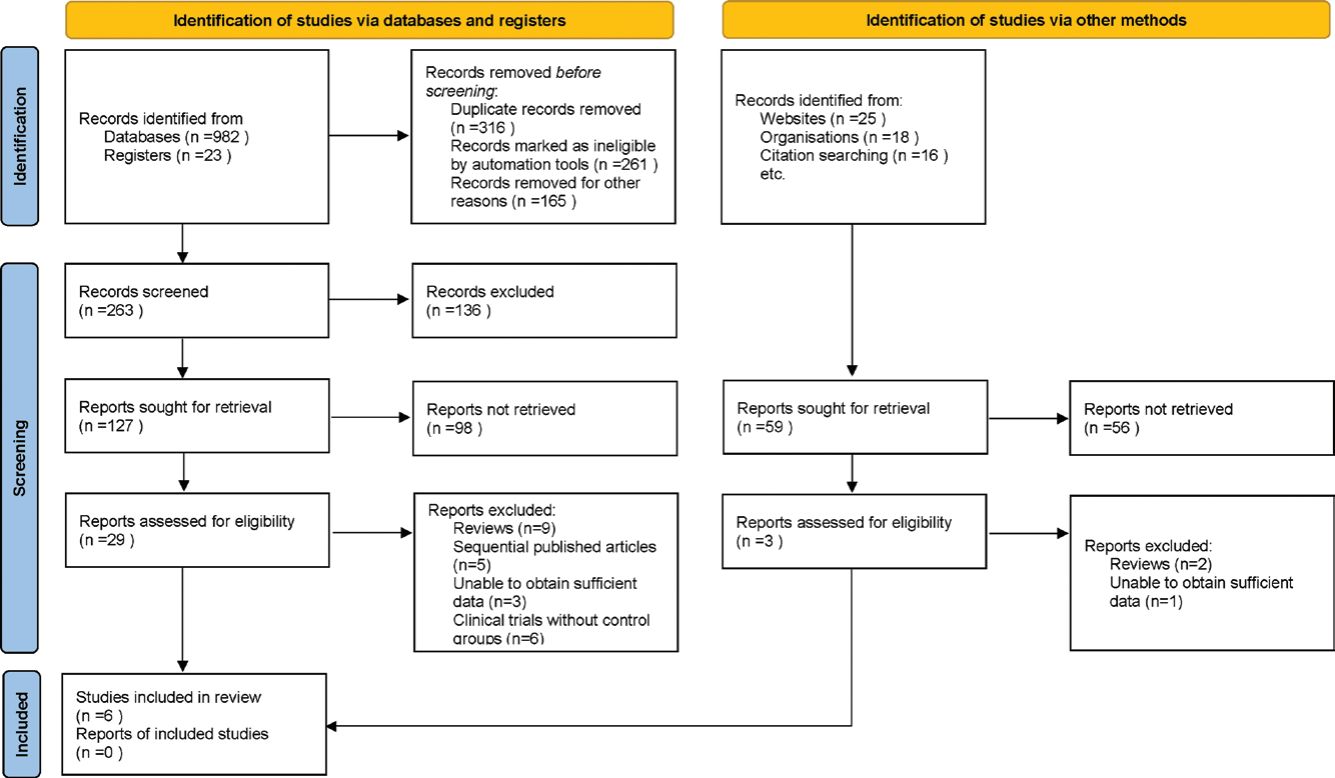
Flowchart depicting the study selection process for inclusion in the meta-analysis.

In the initial phase of our systematic review and meta-analysis, we performed a comprehensive search across several electronic databases, which yielded a preliminary collection of 1064 articles potentially relevant to our study topic. To streamline this initial dataset, a duplication removal algorithm was applied, ensuring that each unique study was represented only once in our pool of resources. Subsequently, we embarked on a rigorous screening process, meticulously evaluating the titles and abstracts of these articles. This evaluation was guided by our predefined inclusion and exclusion criteria, which were meticulously crafted to encompass a variety of factors such as study methodology, demographic characteristics of the study population, clinical outcomes measured, and the overall research quality. Following this preliminary assessment, we narrowed down our selection to 32 articles, which warranted a more detailed examination. This next stage of evaluation involved multiple investigators independently reviewing the full texts of these articles. This thorough examination was pivotal in ensuring an unbiased and comprehensive assessment of each study. During this detailed review process, 26 articles were excluded for specific reasons. These exclusions were categorized as follows: 11 were review articles, 5 were sequentially published works, 4 presented insufficient data for analysis, and 6 were clinical trials lacking control groups. After carefully following our rigorous selection criteria, a total of 6 papers, which were only based on observations, fulfilled all the standards outlined in our study procedure.^[10–15]^ (Fig. 1).

### 3.2. Study characteristics

The meta-analysis incorporated 6 case-control studies, all of which were conducted in Japan over various periods ranging from 2001 to 2016. These studies collectively examined a substantial cohort of 4868 cases. The individual studies, carried out by authors Higashiyama, Matsumura, Mukai, Okada, Sato, and Tsuji, were published between 2010 and 2017. They explored the age demographics of early gastric cancer patients, with mean ages across studies varying from 68.0 to 72.4 years, some of which reported standard deviations, indicating variability within the sample populations. Each study contributed valuable data on the characteristics and outcomes of patients undergoing endoscopic submucosal dissection for early gastric cancer, providing a substantial body of evidence for identifying risk factors associated with postoperative bleeding (Table 1).

**Table 1:** Basic information of included studies.

Author	Year of publication	Study period	Country	Study type	No. of cases	Age (yr)
Higashiyama	2011	2005–2009	Japan	Case-control study	924	68.9
Matsumura	2014	2005–2014	Japan	Case-control study	425	72.1 ± 8.6
Mukai	2012	2007–2010	Japan	Case-control study	161	72.4 ± 8.8
Okada	2011	2005–2008	Japan	Case-control study	582	68.4 ± 9.2
Sato	2017	2001–2016	Japan	Case-control study	2378	72.0 ± 6.9
Tsuji	2010	2007–2009	Japan	Case-control study	398	68.0 ± 10.2

### 3.3. Results of quality assessment

The methodological quality of the included studies in our meta-analysis was rigorously evaluated using the NOS, a standardized tool for assessing the quality of non-randomized studies. The results indicate a generally high quality among the studies: 1 study received a score of 7, indicating good quality, while 2 studies achieved a score of 8, and the remaining three were awarded the maximum score of 9, reflecting excellent quality. Despite the lack of blinding and allocation concealment, which are common limitations in observational studies, there were no identified funding biases across the studies. Additionally, the integrity of the reported outcomes was upheld, with no instances of incomplete outcome data, premature study cessation, or significant baseline imbalances detected. These findings suggest a low risk of bias within the studies, contributing to the reliability of the meta-analysis. The detailed risks of bias and their corresponding ratios have been collated in Table 2 for reference.

**Table 2:** The quality assessment according to Newcastle-Ottawa Scale of each cohort study.

Study	Selection				Comparability	Outcome			Total score
	Representativeness of the exposed cohort	Selection of the non-exposed cohort	Ascertainment of exposure	Demonstration that outcome	Comparability of cohorts	Assessment of outcome	Was follow-up long enough	Adequacy of follow up of cohorts	
Higashiyama	★	★	★	★	★★	★	★	★	9
Matsumura		★	★	★	★★	★	★	★	8
Mukai	★	★	★	★	★★	★	★	★	9
Okada	★	★	★	★	★★	★		★	8
Sato	★	★		★	★	★	★	★	7
Tsuji	★	★	★	★	★★	★	★	★	9

★Each individual asterisk ('★') signifies one point.

### 3.4. Influence of intraoperative ulcer detection on post-endoscopic submucosal dissection bleeding in early gastric cancer

**Figure 2.**
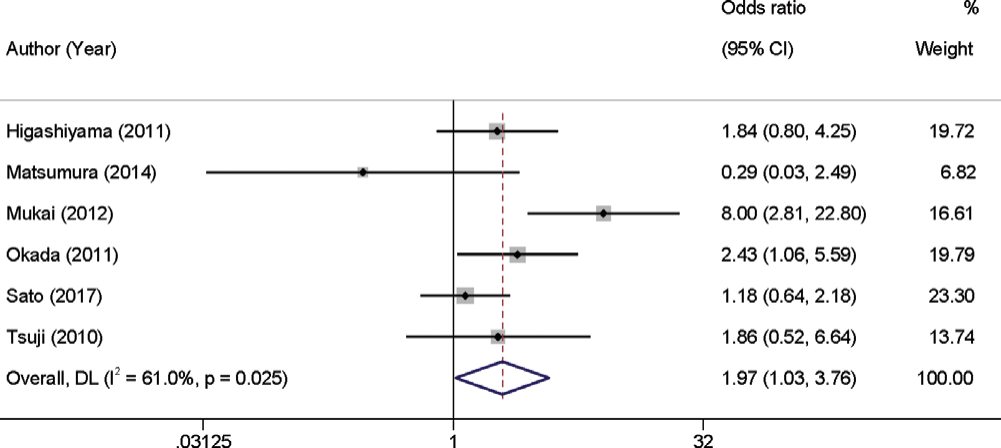
Forest plot illustrating the impact of intraoperative ulcer detection on post-endoscopic submucosal dissection bleeding in early gastric cancer. CI = confidence interval.

Upon a comprehensive review of 6 studies, the impact of intraoperatively detected ulcer positivity on the occurrence of PEB in EGC was examined. The analysis revealed substantial heterogeneity among the studies (*I*^2^ = 61.0%, *P* = .025), prompting the use of a random-effects model for the meta-analysis. The findings demonstrated that the presence of ulcers detected during the surgical procedure was significantly associated with an increased risk of PEB in EGC patients. The odds ratio (OR) was calculated to be 1.97 with a 95% confidence interval (CI) ranging from 1.03 to 3.76, suggesting nearly a 2-fold increase in bleeding risk when ulcers were detected intraoperatively (Fig. 2).

### 3.5. Association of antithrombotic medication use with post-endoscopic submucosal dissection bleeding in early gastric cancer

**Figure 3.**
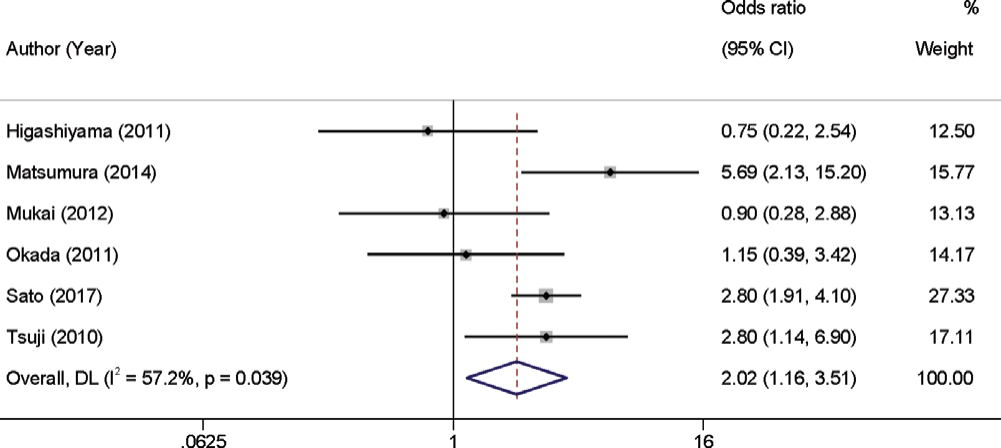
Forest plot demonstrating the relationship between antithrombotic medication use and the risk of postoperative bleeding in early gastric cancer patients. CI = confidence interval.

Our meta-analysis synthesized data from 6 studies examining the impact of antithrombotic medication use on the incidence of PEB in patients with EGC. We observed considerable heterogeneity among the included studies (*I*^2^ = 57.2%, *P* = .039), which necessitated the use of a random-effects model for analysis. The meta-analytic findings revealed a statistically significant increase in the risk of PEB associated with the use of antithrombotic medications during the perioperative period, with an OR of 2.02 and a 95% CI of 1.16 to 3.51 (Fig. 3).

### 3.6. Outcomes of lesion resection size, hypertension, and intraoperative procedure time in early gastric cancer

In this meta-analysis, we also focused on 3 critical aspects: the size of lesion resection, the presence of hypertension, and the duration of the intraoperative procedure in the context of EGC management. Our analysis incorporated studies that provided sufficient data to calculate mean difference and OR for these parameters. For the size of lesion resection, a significant mean difference of 5.16 (95% CI: 2.97–7.98, *P* < .01) was observed, suggesting that larger resections were common among the reviewed cases. The fixed-effect model, indicating low statistical heterogeneity (*I*^2^ = 0.0%), was utilized due to the consistency across study results. Regarding the influence of hypertension on postoperative outcomes, the odds ratio was 0.86 (95% CI: 0.56–1.29, *P* = .23), which did not indicate a significant impact of hypertension on the studied postoperative complications. The fixed-effect model was again applied, reflecting moderate heterogeneity (*I*^2^ = 18.1%) among the included studies. Lastly, the intraoperative procedure time showed a mean difference of 11.69 minutes (95% CI: 1.82–26.20, *P* < .05), indicating that longer surgeries were significantly associated with the studies’ outcomes. A fixed-effect model was chosen due to the absence of heterogeneity (*I*^2^ = 0.0%) in this variable (Table 3).

**Table 3:** Meta-analysis results of 5-HTR1A gene C-1019G polymorphism and antidepressant efficacy in the effective treatment group.

Risk factors	OR/MD	95% CI	*P* value	Model type (heterogeneity)	*P* value (heterogeneity)	*I*^2^ (heterogeneity %)
Intraoperative ulcer detection	1.97	1.03–3.76	<.05	Random	.025	61.0
Antithrombotic medication use	2.02	1.16–3.51	<.01	Random	.039	57.2
Size of lesion resection	5.16	2.97–7.98	<.01	Fixed	.86	0.0
Hypertension	0.86	0.56–1.29	.23	Fixed	.15	18.1
Intraoperative procedure time	11.69	1.82–26.20	<.05	Fixed	.52	0.0

CI = confidence interval, MD = mean difference, OR = odds ratio.

### 3.7. Sensitivity analysis

**Figure 4.**
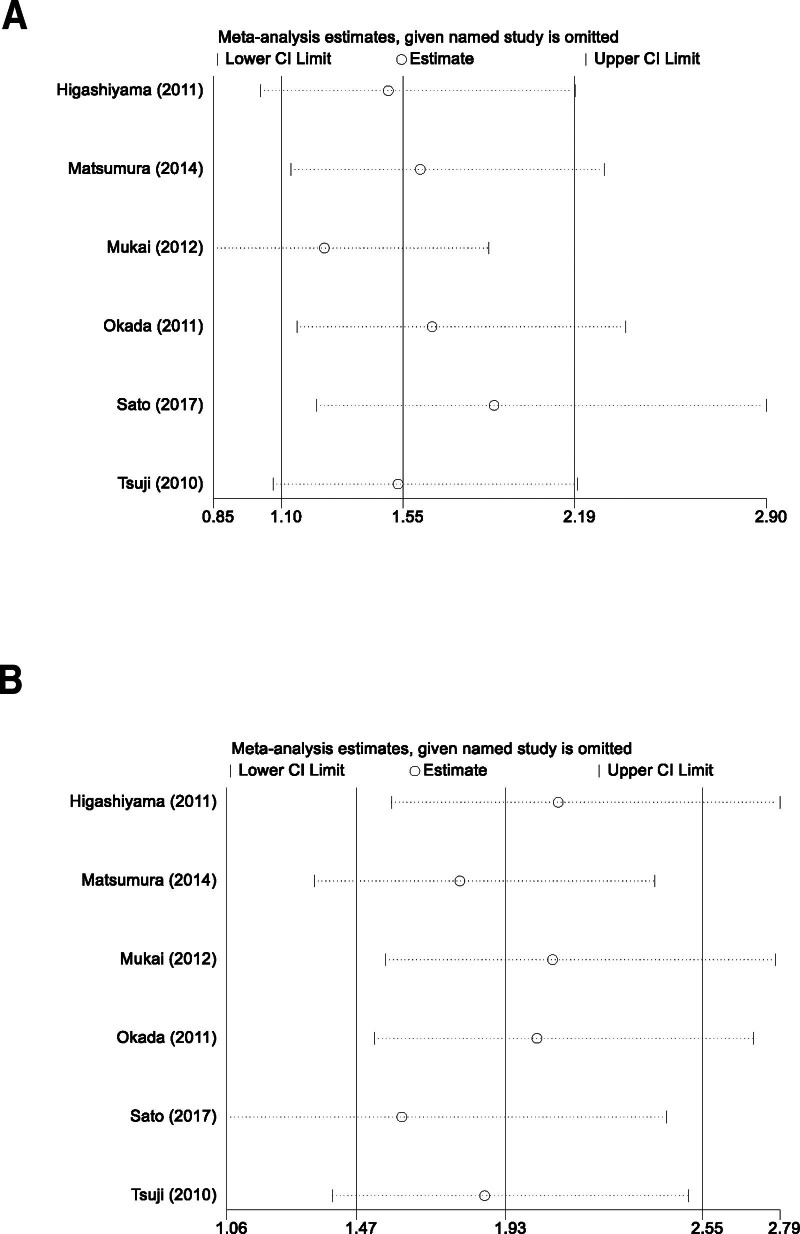
Sensitivity analysis graphs showing the effects of intraoperative ulcer detection (A) and antithrombotic medication use (B) on postoperative bleeding in early gastric cancer. CI = confidence interval.

In light of the observed heterogeneity within the included studies of our meta-analysis, we executed a sensitivity analysis to ascertain the robustness and consistency of our combined effect estimates. This procedure involved a sequential omission approach, where each study was individually removed, and the overall effect size recalculated with the remaining data set. Our meticulous approach to sensitivity analysis underscored that the aggregated results of our meta-analysis were not disproportionately swayed by any single study. This was evidenced by the maintained stability and consistency of the effect estimates across the different iterations of the analysis. Such steadfastness in the pooled results, even after the exclusion of individual studies, bolsters the dependability of the meta-analytic conclusions. The steadfast nature of these results, as depicted in Figure 4, confirms the solidity of our primary conclusions. The sensitivity analysis thereby substantiates the comprehensive synthesis of evidence presented and reinforces the validity of the interpretations derived from our meta-analysis.

### 3.8. Publication bias

**Figure 5.**
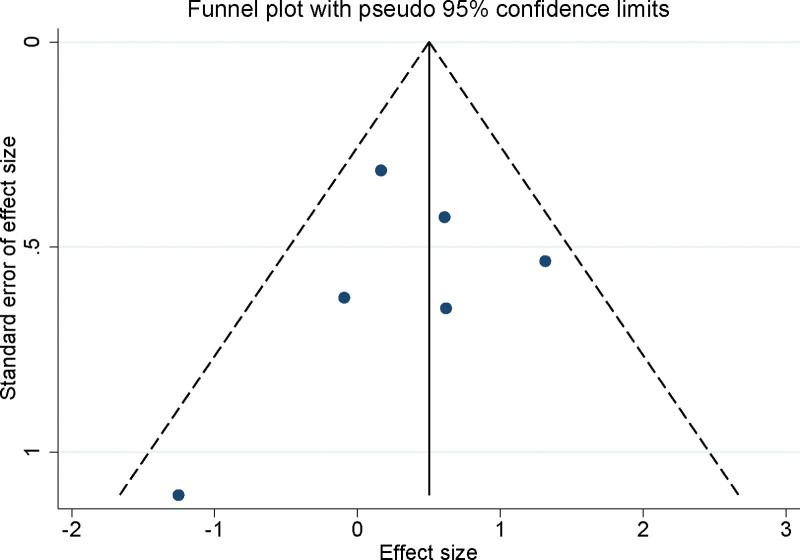
Funnel plot assessing potential publication bias in the studies included in the meta-analysis.

The assessment of potential publication bias in our meta-analysis was meticulously conducted through the construction of funnel plots for the included studies. The visual inspection of these plots revealed a symmetrical distribution, suggesting no evidence of significant publication bias (Fig. 5). Complementing the visual assessment, the Egger’s linear regression test was employed for a more quantitative analysis. This test provided no indication of significant publication bias across the different variables examined in our meta-analysis (*P* > .05 for all examined cases). The absence of detected publication bias on both visual and statistical fronts endorses the credibility of our meta-analysis findings. The combination of funnel plot symmetry and non-significant Egger’s test results consolidates the overall robustness and reliability of the results presented in this meta-analysis.

## 4. Discussion

Our meta-analysis consolidates evidence from pivotal years in the evolution of ESD for EGC, offering a historical and contemporary perspective on risk factors for post-ESD bleeding. By systematically reviewing studies from 2001 to 2016, we identified and quantified key risk factors, including intraoperative ulcer detection, antithrombotic medication use, lesion resection size, and procedural duration. This comprehensive synthesis provides actionable insights for enhancing patient care and refining clinical strategies. The innovation of our study lies in its methodological rigor and the breadth of its analysis, which bridges past and present clinical practices. Through sensitivity analysis and publication bias assessment, we confirm the reliability of our findings, underscoring their significance for current and future endoscopic techniques. This work not only enhances understanding of post-ESD bleeding risks but also lays groundwork for future research in this dynamically evolving field.

The importance of analyzing risk factors for postoperative bleeding in EGC cannot be overstated. Identifying patients at higher risk can facilitate preoperative planning, tailored surgical approaches, and vigilant postoperative care.^[16,17]^ Factors such as lesion size, hypertension, use of antithrombotic drugs, and intraoperative procedure time play crucial roles.^[18]^ By predicting the likelihood of bleeding, clinicians can better inform patients about their individual risks and recovery expectations. This study contributes to the field in several novel and clinically valuable ways. Firstly, it offers a comprehensive analysis of multiple risk factors, providing a broader understanding than previous studies that may have focused on isolated variables.^[19]^ Secondly, the use of a robust meta-analytic approach enhances the reliability of our findings, drawing from a larger pool of data for more generalized conclusions. Finally, the clinical implications of this study are significant. By highlighting key risk factors, it guides clinicians in making more informed decisions, thereby potentially improving patient outcomes in EGC treatment.

The identified association between intraoperative ulcer detection and elevated PEB risk underscores the necessity for heightened clinical vigilance and potential strategic planning in the management of EGC patients with positive ulcer findings. The almost 2-fold increased risk of PEB in patients with detected ulcers during EGC surgery is a clinically relevant finding that may guide preoperative risk assessments and inform intraoperative strategies to mitigate bleeding complications. The observed relationship between the use of antithrombotic medications and an increased risk of PEB in EGC patients is of clinical importance.^[20]^ The elevation in risk highlights the critical need for careful evaluation of the indications for antithrombotic therapy in the context of EGC treatment. Clinicians must judiciously weigh the benefits of antithrombotic medication against the potential for increased bleeding, particularly in the perioperative setting. Strategies may include stricter criteria for medication use, closer monitoring of bleeding signs, and prompt intervention when necessary.

The size of lesion resection is a critical factor in EGC surgery, with our analysis highlighting a trend towards larger resections. This may reflect a clinical preference for ensuring clear margins to prevent recurrence, although it could also raise the risk of postoperative complications such as bleeding or perforation. The absence of heterogeneity suggests a universal approach to lesion resection across the studies. The lack of significant association between hypertension and the outcomes could imply that well-managed hypertension may not be a determining factor in the incidence of complications following EGC surgery. However, the moderate heterogeneity indicates that the role of hypertension may vary depending on other patient or procedural factors. The association between longer intraoperative times and increased risk of postoperative bleeding is significant, suggesting that operational duration may be more intricately linked to lesion characteristics prone to bleeding rather than the extent of tumor involvement. This adjustment emphasizes that longer ESD procedures, necessitated by meticulous dissection required for managing lesions with higher bleeding propensities, underscore the critical need for precise surgical planning tailored to lesion-specific challenges. These findings underscore the importance of meticulous surgical planning and patient management in EGC treatment.^[21]^ They also highlight the need for further research into optimizing surgical techniques and postoperative care to minimize complications, particularly in patients requiring extensive resections or with clinical comorbidities such as hypertension.

In our meta-analysis, we identified several independent risk factors for PEB in EGC, including the use of antithrombotic medications and the extent of lesion resection. While each factor was analyzed for its individual contribution to PEB risk, we propose a nuanced understanding of how these factors may collectively influence patient outcomes in clinical practice. Antithrombotic drugs, essential for thrombosis prevention, also pose a bleeding risk, potentially exacerbated by any concurrent ulcerative conditions. Similarly, larger resections, necessitated by the goal of achieving clear margins, are associated with longer surgical durations, both of which have been independently linked to increased bleeding risks. Our discussion aims to bridge the gap between the independent analysis of risk factors and the complex clinical scenarios where multiple risk factors converge, potentially amplifying the risk of PEB. This approach is rooted in the clinical reality that patients often present with multiple interrelated risk factors, suggesting that the cumulative effect of these factors warrants consideration in the comprehensive management of PEB. To address the concern raised, we emphasize that our interpretation of the cumulative impact of risk factors on PEB risk is an extrapolation intended to enhance clinical insight rather than a direct outcome of statistical interaction analysis. We advocate for a comprehensive clinical assessment that considers not only the individual but also the collective impact of risk factors on PEB, underlining the importance of personalized and meticulous surgical planning, technique adaptation, and vigilant postoperative care to mitigate the overall risk of bleeding. Such a multifaceted approach is crucial for optimizing patient outcomes and care quality in EGC management.

While this study offers valuable insights, it’s important to acknowledge its limitations. Firstly, the inherent heterogeneity among the included studies, despite rigorous analysis, may affect the generalizability of the findings. The variability in study designs, patient populations, and treatment protocols across different studies introduces potential biases. Our sensitivity analysis aimed to identify any single study’s disproportionate influence on the overall meta-analysis findings. However, no individual article was conclusively identified as the sole contributor to the heterogeneity observed in every outcome. This suggests that the heterogeneity likely stems from a combination of factors, including but not limited to, differences in study methodologies, patient demographics, and clinical settings across the included studies. Secondly, the reliance on published literature may lead to publication bias, as studies with negative or inconclusive results are often underrepresented. Additionally, most of the included studies are observational, which inherently limits the ability to establish causation compared to randomized controlled trials. Finally, the lack of data on patient lifestyle factors and genetic predispositions, which could influence postoperative outcomes, was not comprehensively addressed, potentially overlooking critical determinants of postoperative bleeding.

## 5. Conclusions

In managing EGC, key risk factors for PEB include intraoperative ulcer detection, antithrombotic medication use, size of lesion resection, and intraoperative procedure time. These factors do not exist in isolation but interact in a complex manner, cumulatively influencing the risk of PEB. Understanding and addressing these interconnected risks are crucial for improving surgical outcomes and patient safety in EGC treatment.

## Author contributions

**Conceptualization:** Yuanbo Gu.

**Data curation:** Shuchang Zhao.

**Formal analysis:** Yuanbo Gu, Shuchang Zhao.

**Methodology:** Yuanbo Gu.

**Resources:** Shuchang Zhao.

**Software:** Shuchang Zhao.

**Writing – original draft:** Yuanbo Gu.

**Writing – review & editing:** Yuanbo Gu.
